# Vitamin B12 deficiency among adult diabetic patients in Uganda: relation to glycaemic control and haemoglobin concentration

**DOI:** 10.1186/s40200-016-0250-x

**Published:** 2016-07-26

**Authors:** George Patrick Akabwai, Davis Kibirige, Levi Mugenyi, Mark Kaddu, Christopher Opio, Rejani Lalitha, Edrisa Mutebi, Martha Sajatovic

**Affiliations:** 1 Department of Medicine, Makerere University College of Health Sciences, P.O.BOX 7062, Kampala, Uganda; 2 Department of Medicine/Diabetic and Hypertension Clinics, Our Lady of Consolata Hospital Kisubi, Wakiso, Uganda; 3 Infectious Disease Research Collaboration, Kampala, Uganda; 4 Diabetes/Endocrine Unit, Mulago National Referral and Teaching Hospital, Kampala, Uganda; 5 Department of Neurology, Case Western Reserve University and University Hospitals Case Medical Center, Cleveland, OH USA

**Keywords:** Vitamin B12 deficiency, Adults, Black, Individuals with diabetes, Africa, Uganda

## Abstract

**Background:**

Vitamin B12 deficiency is highly prevalent among adult individuals with diabetes yet screening is infrequent in Uganda. There are currently no published data regarding the prevalence of vitamin B12 deficiency and its associated factors among adult individuals with diabetes in sub-Saharan Africa. This study aimed at describing the prevalence and factors associated with vitamin B12 deficiency among this patient population in a resource constrained setting in sub-Saharan Africa.

**Methods:**

In this cross-sectional study, 280 eligible study participants attending the outpatient diabetic clinic at Mulago national referral and teaching hospital in Kampala, Uganda were enrolled. Their socio-demographic, clinical and laboratory data was collected using a pre-tested questionnaire.

**Results:**

The majority of the study participants were female (68.9 %), with a median age of 50 (IQR: 40–58) years. The mean (SD) serum vitamin B12 levels was 472.0 (16.4) pg/ml. The prevalence of vitamin B12 deficiency was 10.7 %. Hemoglobin level < 12 g/dl (AOR 3.38; 95 % CI 1.38–8.32, *p* value = 0.008) and glycated hemoglobin ≥ 7 % (AOR 3.29; 1.44–7.51, *p* value = 0.005) were associated with vitamin B12 deficiency.

**Conclusions:**

Vitamin B12 deficiency is prevalent in approximately 1 in 10 of adult individuals with diabetes in Uganda. We recommend screening for vitamin B12 deficiency among diabetic patients in Uganda especially those with low hemoglobin concentrations and glycated hemoglobin levels ≥ 7 %.

## Background

Vitamin B12 or cobalamin is a water soluble vitamin that plays a very essential role in DNA synthesis, optimal haemopoesis and neurological function [1]. Several studies have unequivocally demonstrated that vitamin B12 deficiency is highly prevalent in adult diabetic patients [2–4]. The most notable risk factor associated with vitamin B12 deficiency in adult individuals with diabetes in clinical practice is long term and high dose metformin therapy [4–6]. Clinically overt and untreated vitamin B12 deficiency has been associated with disabling sensory polyneuropathy which mimics diabetic neuropathy, anaemia, depression, cognitive dysfunction [1, 7, 8].

Despite the above recognised clinical conditions associated with vitamin B12 deficiency and its high frequency in adult individuals with diabetes, there are no global guidelines recommending routine assessment of serum vitamin B12 levels. In sub Saharan Africa (SSA), a region with a high frequency of nutritional deficiencies, there is a dearth of data on the burden and predictors of vitamin B12 deficiency in adult individuals with diabetes.

We therefore sought to determine the prevalence and associated factors of vitamin B12 deficiency in 280 adult individuals with diabetes attending the outpatient diabetic clinic at Mulago national referral and teaching hospital in Kampala, Uganda.

## Methods

Between 1st May and 31st September 2014, we performed a cross sectional study to assess vitamin B12 levels and related clinical factors in the outpatient diabetic clinic of Mulago national referral and teaching hospital, Kampala Uganda. The clinic is run every Wednesday and an average of 80 adult patients is reviewed on each clinic day. Patients enrolled into the study were: ambulatory with documented evidence of DM and had regularly been attending the clinic for at least the past 6 months, aged ≥ 18 years and had offered informed consent. Patients on vitamin B12 replacement therapy or multi vitamin supplementation were excluded.

### Sample size and sampling technique

A consecutive sampling method was used during the recruitment process of the eligible patients until the desired sample size of 280 was obtained. Using the Kish-Leslie formula (1995) of *n* = Z^2^Pq/d^2^ (*n* = Sample size, Z =1.96, the normal value corresponding to the 95 % confidence interval, *P* = 0.22-prevalence of vitamin B12 deficiency of 22 % from a study by Pflipsen M et al. [2] in adult individuals with diabetes, q = 1-p and d = 5 %, the desired precision of estimation), a sample size of a minimum of 264 patients was obtained. We increased the sample size to 280 patients.

### Data collection

A questionnaire in English or Luganda, the commonly spoken local dialect in the region was administered to the study participants. The translation of the questionnaires from English to Luganda was done by someone fluent in the language and adept in questionnaire translation. The translated questionnaires were pre tested to ensure validity of the results to be obtained.

Information obtained included key socio-demographic characteristics like age, gender, employment and education status, clinical signs and symptoms related to vitamin B12 deficiency, past medical and drug history, family history of DM and hypertension and lifestyle and social activities such as alcohol intake and smoking. Participants’ weight in kilograms (kg) and height in metres (m) were measured using a calibrated weight scale and height board respectively and their body mass indexes (BMI) calculated using the formula below; BMI = kg/m^2^.

### Laboratory analysis

Six millilitres of blood were drawn by an experienced laboratory technician and sent for analysis. The complete blood count (CBC) and peripheral film comment were done by an experienced haematologist in the haematology laboratory of Mulago hospital. Serum vitamin B12 levels, the outcome variable, glycated haemoglobin (HbA1c), fasting lipid profile, renal and liver function tests were analysed from the clinical chemistry laboratory of Mulago hospital using the Roche diagnostics Elecsys© immunoassays. The main outcome of this study was vitamin B12 deficiency defined as serum vitamin B12 serum levels < 200 pg/ml. Borderline vitamin B12 deficiency and normal vitamin B12 levels were defined as vitamin B12 levels between 200 and 300 pg/ml and >300 pg/ml respectively.

### Statistical analysis

Pre-coded data were entered using EPI-DATA 3.1 and were exported to statistical package, STATA 10, for statistical analysis with the help of a statistician. Age was presented as median as this was not normally distributed and grouped onto age categories for further analysis. Categorical variables were analyzed and presented as frequencies and percentages. They were compared using the chi-square and Fisher’s exact tests as appropriate. At bi-variable analysis, variables with a *p* vaues ≤ 0.2 were subjected to multivariable analysis using a logistic regression and model building done using a likelihood ratio test.

The prevalence of vitamin B12 deficiency was expressed as the proportion of study participants with serum vitamin B12 levels < 200 pg/ml divided by the total number of participants enrolled into the study. A *p*-value of 0.05 was considered statistically significant.

## Results

### Socio demographic and clinical characteristics of study participants

Of the 280 study participants enrolled into the study, there was a female predominance (193, 68.9 %). The median age (IQR) for the study participants was 50 (40–58) years. The majority of the study participants were urban dwellers (175, 62.5 %), unemployed (147, 52.7 %) and had attained primary level education (137, 48.9 %) (summarised in Table 1).

**Table 1:** Demographic characteristics of the study participants (*N* = 280)

Characteristic		*n* (%)
Age, median (IQR)		50 (18)
Gender, *n* (%)	Female	193 (68.9)
Address, *n* (%)	Rural	105 (37.5)
	Urban	175 (62.5)
Education level, *n* (%)	None	31 (11.1)
	Primary	137 (48.9)
	Secondary	100 (35.7)
	Post-secondary	12 (4.3)
Religion, *n* (%)	Muslim	72 (25.8)
	Catholic	86 (30.8)
	Anglican	86 (30.8)
	Born again	35 (12.5)
Occupation, *n* (%)	Employed	133 (47.5)
	Unemployed	147 (52.5)
Marital status, *n* (%)	Married	156 (55.7)
	Cohabiting	10 (3.6)
	Single	28 (10.0)
	Divorced	41 (14.6)
	Widow/widowed	45 (16.1)

The most frequent clinical symptoms were paraesthesias (227, 81.1 %), forgetfulness (24, 8.57 %) and tiredness even after light exertion (14, 5 %). Others included irritability (12, 4.3 %) and gait abnormality (11, 3.9 %). Clinical pallor of the mucous membranes and palmar pigmentation were observed in only 8 (2.86 %) and 3 (1.1 %) study participants respectively. Hypertension, prior use of proton pump inhibitors (PPIs) and HIV infection were documented in 188 (67 %), 73 (26 %) and 17 (6.1 %) study participants respectively. Family history of hypertension and diabetes was reported in 200 (71.4 %) and 187 (66.8 %) participants respectively. Most participants were type 2 diabetics (224, 81.5 %).

### Vitamin B12 status of the study participants

The mean (SD) serum vitamin B12 level of the study participants was 472 (16.4) pg/ml. Vitamin B12 deficiency, borderline vitamin B12 deficiency and normal vitamin B12 levels were documented in 30 (10.7 %), 42 (15 %) and 208 (74.3 %) participants respectively. A total of 72 (25.7 %) participants had suboptimal vitamin B12 levels, defined as vitamin B 12 levels < 300 pg/ml. All these study participants had never had a serum vitamin B12 measurement performed before.

### Socio-demographic and clinical characteristics of the study participants at bivariable analysis

At bivariable analysis, neither socio-demographic nor clinical characteristics were associated with vitamin B 12 deficiency. (Tables 2 and 3 summarizes the socio-demographic and clinical characteristics of the study participants in association with vitamin B12 levels at bivariable analysis).

**Table 2:** Vitamin B12 deficiency in relation to socio-demographic characteristics at bivariable analysis

Characteristic		<200 pg/ml *N* = 30 (%)	≥200 pg/ml *N* = 250 (%)	*P* value
Age in years	<40	4 (13.3)	72 (28.8)	0.137
	40–60	18 (60.0)	136 (54.4)	
	>60	8 (26.7)	42 (16.8)	
Sex	Male Female	6 (20.0) 24 (80.0)	81 (32.4) 169 (67.6)	0.166
Address	Rural Urban	9 (30.0) 21 (70.0)	96 (38.4) 154 (61.6)	0.369
Education level	None/primary Post primary	19 (63.3) 11 (36.7)	149 (59.6) 101 (40.4)	0.693
Religion	Muslim Catholic Anglican Born-again	7 (23.3) 11 (36.7) 9 (30.0) 3 (10.0)	65 (26.1) 75 (30.1) 77 (30.9) 32 (12.9)	0.889

**Table 3:** Vitamin B12 deficiency in relation to clinical characteristics at bivariable analysis

Characteristic		<200 pg/ml, *n* (%)	≥200 pg/ml, *n* (%)	*P* value
Paraesthesias	No	4 (13.3)	49 (19.6)	0.621
	Yes	26 (86.7)	201 (80.4)	
Forgetfulness	No	28 (93.3)	228 (91.2)	>0.999
	Yes	2 (6.7)	22 (8.8)	
Tiredness	No	30 (100)	236 (94.4)	0.376
	Yes	0 (0.0)	14 (5.6)	
Gait	No	27 (90.0)	242 (96.8)	0.101
	Yes	3 (10.0)	8 (3.2)	
Irritability	No	29 (96.7)	238 (95.6)	>0.999
	Yes	1 (3.3)	11 (4.4)	
Pallor	No	28 (93.3)	244 (98.6)	0.207
	Yes	2 (6.7)	6 (2.4)	
Hypertension	No	9 (30.0)	82 (32.8)	0.757
	Yes	21 (70.0)	168 (67.2)	
HIV	No	28 (93.3)	235 (94.0)	0.701
	Yes	2 (6.7)	15 (6.0)	
Family history of DM	No	12 (40.0)	81 (32.4)	0.404
	Yes	18 (60.0)	169 (67.6)	
Family history of HT	Yes	10 (33.3)	68 (27.4)	0.496
	No	20 (66.7)	180 (72.6)	
Current medication	Metformin alone	7 (23.3)	41 (16.4)	0.726
	Metformin & SU	12 (40.0)	78 (31.2)	
	Metformin & SU & Pio	1 (3.3)	9 (3.6)	
	Insulin alone	6 (20.0)	68 (27.2)	
	Insulin & metformin	4 (13.3)	50 (20.0)	
	Statins	0 (0.0)	4 (1.6)	
Age at diagnosis (years)	<40	8 (26.7)	98 (39.2)	0.144
	40–60	21 (70.0)	129 (51.6)	
	>60	1 (3.3)	23 (9.2)	
Duration since diagnosed (years)	<2	7 (23.3)	59 (23.6)	0.234
	2–4	6 (20.0)	85 (34.0)	
	5–7	3 (10.0)	32 (12.8)	
	>7	14 (46.7)	74 (29.6)	
BMI Kg/m^2^	<18.5	2 (6.7)	18 (7.2)	0.939
	18.5–24.9	10 (33.3)	96 (38.6)	
	25.0–29.9	10 (33.3)	71 (28.5)	
	≥30.0	8 (26.7)	64 (25.7)	
Blood pressure (mmHg)	≤140/80	11 (36.7)	115 (46.0)	0.332
	>140/80	19 (63.3)	135 (54.0)	
Metformin alone (*N* = 48)	≤3 years	4 (57.1)	27 (65.9)	0.657
	>3 years	3 (42.9)	14 (34.2)	
Metformin & SU (*N* = 90)	≤3 years	5 (41.7)	41 (52.6)	0.484
	>3 years	7 (58.3)	37 (47.4)	
Insulin alone (*N* = 74)	≤3 years	5 (83.3)	32 (47.1)	0.124
	>3 years	1 (16.7)	36 (52.9)	

*Abbreviations*: *DM* diabetes mellitus, *HT* hypertension, *BMI* body mass index, *SU* sulphonylurea, *Pio* pioglitazone, *PPIs* proton pump inhibitors

### Laboratory findings of the study participants at bivariable analysis

A statistically significant association with vitamin B12 deficiency was observed with hemoglobin concentration < 12 g/dl (*p* = 0.001), WBC counts > 4000 cells/mm^3^ (*p* = 0.017) and glycated hemoglobin ≥ 7 % (*p* = 0.001). Paradoxically, a high mean cell volume (MCV) ≥ 100 was infrequent in participants with vitamin B12 deficiency (1, 3.4 %). The majority had normal MCV levels (18, 62.1 %). No study participant with vitamin B12 deficiency had the classical findings of macrocytosis, ovalocytosis and hyper segmented WBCs on peripheral blood film assessment (Table 4 summarizes the laboratory findings of the study participants in association with vitamin B12 levels at bivariable analysis).

**Table 4:** Vitamin B12 deficiency in relation to laboratory findings at bivariable analysis

Characteristic		<200 pg/ml *n* (%)	≥200 pg/ml *n* (%)	*P* value
Haemoglobin, g/dl	≥12.0	18 (60.0)	211 (84.4)	0.001
	<12.0	12 (40.0)	39 (15.6)	–
WBC count, cells/mm^3^	<4	5 (16.7)	13 (5.3)	0.017
	≥4	25 (83.3)	234 (94.7)	–
Mean cell volume, fl	<79.9	10 (34.5)	63 (25.6)	0.186
	80–99.9	18 (62.1)	181 (73.3)	–
	≥100	1 (3.4)	3 (1.2)	–
Red cell distribution width, %	<17	29 (96.7)	246 (98.8)	0.367
	≥17	1 (3.3)	3 (1.2)	–
FBS level, mmol/l	<7	9 (30.0)	62 (25.2)	0.570
	≥7	21 (70.0)	184 (74.8)	–
HbA1c, %	<7	16 (53.3)	59 (23.7)	0.001
	≥7	14 (46.7)	190 (76.3)	–
LDLC, mmol/l	<2.6	8 (26.7)	59 (24.1)	0.756
	≥2.6	22 (73.3)	186 (75.9)	–
TGL, mmol/l	<1.7	16 (53.3)	106 (43.3)	0.295
	≥1.7	14 (46.7)	139 (56.7)	–
Total cholesterol, mmol/l	<5	14 (46.7)	128 (51.6)	0.594
	≥5	16 (53.3)	119 (48.2)	–
HDLC, mmol/l	<1	11 (36.7)	62 (25.3)	0.183
	≥1	19 (63.3)	183 (74.7)	–
Serum creatinine, μmol/l	<125	28 (93.3)	236 (96.3)	0.342
	≥125	2 (6.7)	9 (3.7)	–
Serum urea, mmol/l	<6.5	28 (96.6)	219 (90.9)	0.486
	≥6.5	1 (3.5)	22 (9.1)	–
ALT, U/L	<60	30 (100)	245 (99.6)	>0.999
	≥60	0 (0)	1 (0.4)	–
AST, U/L	<60	27 (93.1)	243 (99.2)	0.057
	≥60	2 (6.9)	2 (0.8)	–
Albumin, g/L	<35	2 (6.7)	15 (6.1)	>0.999
	≥35	28 (93.3)	232 (93.9)	–
HIV serology	No	28 (93.3)	232 (94.3)	0.688
	Yes	2 (6.7)	14 (5.7)	–

*Abbreviations*: *WBC* white blood cell, *FBS* fasting blood sugar, *HbA1c* glycated haemoglobin, *LDLC* low density lipoprotein cholesterol, *TGL* triglycerides, *HDLC* high density lipoprotein cholesterol, *ALT* alanine transaminases, *AST* aspartate transaminases, *HIV* human immunodeficiency virus

### Factors significantly associated with vitamin B12 levels at multivariable analysis

At multivariable analysis, the statistically significant factors associated with vitamin B12 deficiency were: hemoglobin concentration < 12 g/dl (adjusted OR-3.38; 95 % CI 1.38–8.31, *p* vaue = 0.008) and glycated hemoglobin ≥ 7 % (adjusted OR-3.29; 95 % CI 1.44–7.51, *p* vaue = 0.005), as summarised in Table 5.

**Table 5:** Unadjusted and adjusted odds ratios for predictors of B12 deficiency using a logistic regression

	Unadjusted		Adjusted	
	OR (95 % CI)	*p* value	OR (95 % CI)	*p* value
Haemoglobin (g/dl)				
≥ 12.0	1	–	1	–
< 12.0	3.61 (1.61–8.08)	0.002	3.38 (1.38–8.31)	0.008
HbA1c^a^				
< 7	1	–	1	–
≥ 7	3.68 (1.69–7.98)	0.001	3.29 (1.44–7.51)	0.005
WBC^b^ count (/mm^3^)				
< 4	1	–	1	–
4–11	0.28 (0.09–0.84)	0.024	0.37 (0.10–1.38)	0.138

Controlled for age and gender variables

^a^HbA1c-glycated haemoglobin

^b^WBC-White blood cell

## Discussion

### Burden of vitamin B12 deficiency

To our knowledge, this is the first study to describe the burden of vitamin B12 deficiency and its associated factors in adult individuals with diabetes in sub Saharan Africa, a region with a high frequency of nutritional deficiencies [9, 10]. In this cross sectional study, we report a prevalence of vitamin B12 deficiency of 10.7 % among adult individuals with diabetes attending the outpatient diabetic clinic of Mulago national referral and teaching hospital in Uganda. Haemoglobin concentration < 12 g/dl and glycated hemoglobin ≥ 7 % were noted to be independently associated with vitamin B12 deficiency.

Varied prevalence of vitamin B12 deficiency in adult individuals with diabetes has been described by studies performed in different countries outside Africa [2–4, 6, 11–13]. In comparison to the findings in our study, higher frequencies of vitamin B12 deficiency have been reported in U.S. and European studies. A cross sectional study by Pflipsen et al. involving 203 outpatient type 2 diabetic patients at a large military primary care clinic in USA documented a prevalence of definite vitamin B12 deficiency of 22 % [2]. Similar studies done in Europe have reported prevalence of about 27 % [12, 13].

The apparently higher prevalence in the European and American studies compared to ours was likely at least in part because of differential cut-offs for categorizing deficient vitamin B12 levels. The study by Pflipsen et al. performed in the USA defined definite vitamin B12 deficiency as serum vitamin B12 concentrations of <100 pg/ml or elevated serum methylmalonic acid of >243 nmol/L or homocysteine concentrations of >11.9 nmol/L if serum vitamin B12 concentrations were between 100 and 350 pg/mL [2]. Therefore, the prevalence in this study would have been higher using a similar cut off < 200 pg/ml.

Differential prevalence rates of vitamin B12 deficiency could also be due to genetic influences and differences in dietary intake (low dietary intake of foods rich in vitamin B12 like meat).

Comparable prevalence of vitamin B12 deficiency of 9.5–14.2 % has been reported in similar studies among South Korean [3, 11] and Indian [12] adult individuals with diabetes. However, despite the comparable prevalence noted in the South Korean studies [3, 11], different study definitions of vitamin B12 deficiency were used (serum vitamin B12 levels ≤300 pg/mL without folate deficiency).

In Uganda, the burden of vitamin B12 deficiency has previously been studied in 2 patient populations: psychiatric patients who had a prevalence rate of 28.6 % [14] and outpatient HIV infected patients who had a prevalence rate of 10.3 % [15]. The study in psychiatric patients defined vitamin B12 deficiency as serum vitamin B12 levels <240 pg/ml which likely accounted for the apparently higher rates compared to our study of diabetic outpatients.

### Factors associated with vitamin B12 deficiency at bivariable and multivariable analysis

At bivariable analysis, a statistically significant association with vitamin B12 deficiency was observed with hemoglobin level < 12 g/dl, glycated hemoglobin ≥ 7 % and WBC count. However, at multivariable analysis, hemoglobin level and glycated hemoglobin retained statistical significance.

Hematological derangements presenting either as a lone cytopenia or pancytopenia are a common finding in patients with vitamin B12 deficiency. Vitamin B12 is an essential micronutrient required in DNA synthesis, cellular repair and optimal haemopoesis together with other micronutrients like folate and iron. In addition to pancytopenia, other overt hematological findings like macrocytic red blood cells (mean cell volume [MCV] > 100 fl) with/without anemia, ovalocytes and hyper segmented white blood cells (i.e. >5 % of neutrophils with ≥5 lobes) are also very frequent. However, these hematological derangements are usually preceded by neurological manifestations like irritability, gait disturbances and paraesthesias [1, 16], which were highly prevalent in our study population.

Contradictorily, raised MCV levels were uncommon among the participants with vitamin B12 deficiency in our study. This could be due to the high prevalence of co-existing iron deficiency anemia in SSA arising from poor dietary intake and helminth infestation [10]. We did not however perform a comprehensive dietary assessment, serum ferritin level measurement and stool examination for ova and cysts in this study.

### Glycated hemoglobin and vitamin B12 deficiency

Glycated haemoglobin level ≥ 7 % which reflects suboptimal glucose control in adult individuals with diabetes was associated with vitamin B12 deficiency in this study population. Compelling evidence from several studies have associated vitamin B12 deficiency and accompanying hyperhomocysteinemia with increased oxidative stress and insulin resistance which collectively result into suboptimal glycaemic control [17–19]. Studies have also demonstrated that low maternal vitamin B12 levels in early pregnancy are often associated with offspring insulin resistance and future DM [20, 21]. The speculative mechanism to explain this is epigenetic modifications due to absence of methionine, a ubiquitous 1-carbon donor to numerous methylation reactions, including DNA methylation. This contributes to elevation in insulin resistance as has been suggested in animal models [22].

Other studies have demonstrated a close association between vitamin B 12 deficiency and long term use and high dose metformin therapy [3–6, 11, 13, 23]. Metformin has been shown to reduce calcium bioavailability which affects ileal vitamin B12 absorption [1, 24]. In our study, this association was not observed probably because there were few patients on metformin therapy.

### Study limitations

The diagnosis of vitamin B12 deficiency is best assessed using levels of serum homocysteine and methylmalonic acid which helps identify patients with borderline (low normal) levels. Unfortunately, we were unable to perform these tests because they are not readily available in Uganda. The study was performed from a single study site or urban hospital based. Therefore, these results cannot be extrapolated to other hospitals and the general non-clinical population at a national level. Finally, the cross-sectional design could not establish a cause-effect relationship between vitamin B12 deficiency and other clinical factors such as diabetes control. Even though more numbers of study participants were enrolled than the desired number, the study still faces a limitation of small sample size particularly in some of the individual cell sizes.

## Conclusions

Vitamin B12 deficiency occurs in approximately 1 in 10 of adult ambulatory individuals with diabetes receiving care in a Ugandan national referral hospital. Low hemoglobin concentrations < 12 g/dl and elevated glycated hemoglobin levels ≥ 7 % (suboptimal glycaemic control) were associated with vitamin B12 deficiency. Elevated MCV levels and red blood cell changes like macrocytosis and hyper segmented WBCs were not as prevalent as expected in patients with vitamin B12 deficiency. Given the relatively high rates and known potential problems associated with vitamin B12 deficiency, we recommend screening for all adult individuals with diabetes, especially in those with suboptimal diabetes control and anemia. We also recommend a prospective representative multi-centre study to assess the prevalence of vitamin B12 deficiency in individuals without diabetes as controls compared to those with diabetes and to examine the effect of vitamin B12 deficiency on optimal glycaemic control and worsening of insulin resistance among individuals with diabetes.
